# Time in the Therapeutic Range (TTR): An Overly Simplified Conundrum

**DOI:** 10.19102/icrm.2017.080302

**Published:** 2017-03-15

**Authors:** James A. Reiffel

**Affiliations:** ^1^Professor Emeritus of Medicine, Columbia University

**Keywords:** Anticoagulation, atrial fibrillation, INR, prothrombin, warfarin

## Abstract

Anticoagulation with warfarin has been used for over 60 years; however, its administration is fraught with difficulty since many factors can affect warfarin dosing. The concept of time in therapeutic range (TTR) has become a popular way to report warfarin management and compare anticoagulation management across clinical trials. However, TTR is a much more complex concept than most clinicians and trialists recognize. This manuscript attempts to discuss some of the important factors underlying this complexity and presents a suggestion to improve reports on TTR.

## Introduction

Anticoagulation with warfarin has been relied upon for over 60 years. When used properly, it is highly effective for reducing stroke and systemic embolism (SSE) in at-risk patients with atrial fibrillation (AF) or mechanical heart valves, as well as for treating and preventing venous thromboembolism (VTE).^1^ However, warfarin is a difficult drug to use because of its many dosing choices; possible dietary, drug and herbal interactions; and its effect on bleeding. Given these challenges, many physicians and patients try to find reasons to avoid it.

To achieve effective and safe dosing, warfarin administration is typically coupled to blood test monitoring including prothrombin time (PT) measurement. The results are reported as an international normalized ratio (INR) so that they are consistently understandable, regardless of the laboratory methodology used. The INR range that most effectively balances the risk of SEE or VTE with the risk of bleeding is 2.0 to 3.0, except in the case of patients with mechanical valves and some Asian populations, for whom slightly higher and lower target ranges are warranted, respectively.^2,3^

Unfortunately, most patients’ INR vary across time. Dietary fluctuations; changes in the pharmacy-dispensed formulation; the introduction, cessation, or alteration of the dosage of one or more concomitant medications, supplements, or over-the-counter agents; changes in bowel flora or function; comorbid or chronic diseases; and the effects of drugs like antibiotics or nonsteroidal anti-inflammatory drugs can all affect the PT, and thus, the required dose of warfarin.^4^

## Methods and results

As a means of assessing the adequacy of anticoagulation with warfarin, the time in the therapeutic range (TTR) method has become a common reportable measure, especially in recent clinical trials.^5^ TTR represents the percentage of time in which the INR remains in the 2.0 to 3.0 target range across time. Simplistically, the TTR should be an easily understandable and easily calculated number. At first glance, TTR might simply be the number of INR values in the target range (numerator) over the total number of INR values measured (denominator); that is, the fraction of INRs in the target range. This has been one of three general approaches to its calculation, both in the cases of individual patients and for a cohort of patients in a trial. The other two approaches are the cross-sectional and linear interpolation methods (see below).^6,7^ However, consider for a moment the following questions: 1) should one use the PTs measured in the first week or two before the warfarin effect is stabilized, recording information obtained before INR reached the target range when calculating the TTR; 2) what about INR results during planned temporary discontinuations of warfarin, such as during surgery, should these be included or excluded when calculating the TTR; and 3) how should one account for differences in the frequency of INR measurements? Ideally, the INR should be measured daily, but this is almost never the case. Rather, measurements are made intermittently: either weekly, biweekly, or monthly. Are we really able to compare the average TTR in patients with only a few measurements versus those with many in the same time frame and, how should we handle non-measured fluctuations that can occur between measurements? This is of particular concern in large multicenter trials, where the frequency of INR determinations varied significantly across countries or regions within countries.^8–11^ Hence, the consideration of multiple geographical regions, such as the United States, North America, western Europe, eastern Europe, South America, the far east and/or Australia will involve different frequencies of INR assessments. This is an important issue when trying to compare TTRs reported for the ARISTOTLE, ENGAGE AF, RE-LY and ROCKET AF trials for which geographical locations varied substantially.^8–11^

The second calculation approach that has been used for populations but not individual patients is the cross-section-of-the-files methodology. With this approach, the number of INRs within the target range for all patients is divided by the total number of INRs completed with respect to all patients at the same point in time (such as at the midpoint of a date interval ± 7 days), using the last INR prior to the chosen date.^6,7^ This provides a somewhat static picture, unless it is repeated over time to compute an average. This approach can be useful to estimate the TTR for a group of patients not being managed by a computerized anticoagulation database, though a limitation of this tactic is diminished accuracy due to the random variation of INR values.

The third approach is that first proposed by Rosendaal et al.^12^ It uses linear interpolation and requires a computerized system for calculation due to its complexity. INR values are assigned to days without measured INRs via the drawing of a linear plot from the last measured INR to the next measured INR. Using values that are represented by this line, a value is assigned to each day. Then, all days with measured or assigned values are used to calculate the time in therapeutic range (TIR). This is not generally useful for individual patients, but as originally described or with minor modifications, it has been successfully used the recent novel oral anticoagulant (NOAC) versus warfarin pivotal AF trials.^8–11^ The referred-to modification is to exclude INR values during therapy initiation, such as for the first week, or during interruptions in which the INR values do not represent stable periods of warfarin administration. Such modifications were used specifically in ARISTOTLE and RE-LY.^8–11^

In general practice in the United States, the reported TTR has varied from ∽25% to 65% across physician specialties, geographical regions and population groups, with an average of approximately 50% to 55%, even in the current era.^13,14^ Notably, however, the TTR calculation method has not been uniform in these comparisons. In the ninth National Conference on Anticoagulant Therapy in 2007, approximately 75% of attendees reported measuring the TTR as a quality indicator for their anticoagulation clinics. However, there was a general unawareness that the method used to measure TTR can affect the results. Approximately 65% used the fraction of INRs in range, 15% relied upon the linear interpolation analysis and 10% employed the cross-section-of-the-files method.^6^

In recent major clinical trials, the numbers have been higher, with mean and median values often in the mid- to high 60% range.^8–11^ In the case of the four recent NOAC versus warfarin trials in AF, the respective mean and median TTR values were 62.2% and 66.6% in ARISTOTLE; 64.9% and 68.4% in ENGAGE AF; 55% and 58% in ROCKET AF; and a mean of 64% in RE-LY, with no median reported.^8–11^

Regardless of the method used, however, the question of whether we can truly compare TTRs across clinical trials still remains, especially given that the issues raised above (i.e., discrepancies in the frequency of measurements and/or in the handling of measurements at the initiation of therapy or during interruption) are handled differently, even when a single TTR calculation method such as the Rosendaal approach was used.^15^ Interestingly, Singer et al. suggested a modification of the Rosendaal approach that makes assumptions about alterations in INR that result from changes in warfarin doses between actual INR rechecks.^16^ However, even this approach does not satisfy most of the concerns raised above. Thus, there are no clear answers to these questions, rendering the interpretation of measured TTRs both complex and somewhat uncertain, making intertrial comparisons hazardous.

Finally, we also need to consider two important numbers— namely, the percentage of INRs that are too low and too high (<2.0 and >3.0, respectively). Simply reporting the TTR does not tell us about these important considerations. If each of the two trials had a mean TTR of 66%, but 30% of the INRs were < 2.0 and 2% were > 3.0 in the first and the second had 2% below 2.1 and 30% above 3.0, then the concerns and possible consequences would be quite different. There could be increases in thromboembolism and bleeding risks in the former and latter, respectively. One could even speculate as to whether differences in the NOAC versus warfarin bleeding rates among the recent pivotal trials might in part be due to such (unreported) factors. It also might explain why the correlation of outcome events versus the quartiles of INR values within a trial, such as in ROCKET AF, have been less than might logically have been anticipated.^1,6^

Accordingly, I suggest that rather than reporting the TTR alone, that we instead consider reporting TTR-F, M%, N, R, X_1_% and X_2_%, where F is the average time between INR checks, M is the mean of all INRs, N is the number of INR measurements, R is the range of INR values, X_1_ is the percentage of INRs <2.0 and X_2_ is the percentage of INRs > 3.0. Across trials, if significant differences were to exist among these numbers, they could be important in understanding and comparing the reported efficacy and bleeding rates in the trials, despite similar mean TTR values. Consider, for example, the values in **Table 1**.

With respect to the data in **Table 1**, both trials A and B have numerically averaged TTRs of 72.5%, but trial A should have a lower risk of bleeding than trial B, while trial A might have a higher risk of thromboembolism due to the difference in the INRs being out of range. One cannot tell this when interpreting the results of these trials based only on the TTR. Furthermore, trial C appears to have a TTR <50%, but this includes low INRs from the first week (as is done with the non-modified Rosendaal method). Finally, if linear interpolation was used in an attempt to provide a number for each trial day, as per Rosendaal, and as has been typical in recent clinical trials, the calculated INRs will differ from the numbers above by some amount, since a value would be assigned via interpolation for each day in the trial, and all of these would be used to calculate the overall TTR. Given the values for F in each trial, the interpolated (i.e., non-measured) INRs would be utilized the most in trial C and the least in trial A.

## Discussion and conclusions

The points discussed above and the examples provided in **Table 1** illustrate some of the complexities involved in calculating and interpreting TTR values; some of the limitations of TTR in making comparative judgements about the quality of warfarin treatment across centers, populations and/or trials; and concerns that we should have about the usually non-reported percentage of time that the INR is outside the target range. Additionally, I do not know of any direct outcome study showing that a particular method of calculating TTR is the best one, although when comparisons have been made within single sets of data, the TTR found by the Rosendaal method has generally been somewhat lower than that determined by the other two approaches.^6,7^ Consequently, I believe that clinical trialists and the Food and Drug Administration need to consider these factors when assessing and comparing warfarin trials: namely, that some means of reporting these pertinent factors beyond just the TTR must become routine, and that clinicians need to understand them when extrapolating clinical trial results to their own practices. Finally, although I proposed above that TTRs be reported with additional information, this concept has not yet been applied to clinical practice, so there are no data to support that it is effectively superior in correlation with either efficacy or safety outcome results.

## Figures and Tables

**Table 1:** TTR-F, M%, N, R, X_1_ and X_2_

**Trial A** (2 years): 2.5, 1.7, 1.7, 2.6, 2.9, 2.1, 1.7, 2.8, 2.7, 2.9, 1.6, 3.0, 2.5, 2.5, 2.8, 1.9, 2.4, 2.6, 2.3, 3.0, 2.7, 1.7, 1.7, 2.6, 2.9, 2.1, 1.7. 2.8, 2.7, 2.9, 1.6, 3.2, 2.5, 2.5, 2.8, 1.9, 2.4, 2.6, 2.3, 3.0 TTR (by numerical average) = 72.5%, F = 15 days (data not shown), M = 2.5, N = 40, R = 1.6–3.2, X_1_ = 25% and X_2_ = 2.5%
**Trial B** (2 years): 2.8, 2.5, 2.6, 2.3, 3.2, 3.3, 2.8, 2.2, 2.7, 3.4, 2.3, 2.6, 3.5, 3.0, 2.9, 2.7, 2.7, 2.4, 2.9, 3.1, 3.4, 2.9, 2.2, 1.8, 2.4, 2.9, 3.3, 3.5, 2.8, 2.7, 2.4, 2.9, 3.5, 3.0, 2.8, 3.2, 2.8, 2.4, 2.9, 3.0 TTR (by numerical average) = 72.8%, F = 20 days (data not shown), M = 2.8, N = 40, R = 1.8–3.5, X_1_ = 2.5% and X_2_ = 25%
**Trial C** (18 months): 1.1, 1.3, 1.7, 2.1, 2.4, 1.9, 2.7, 3.3, 3.1, 2.5, 2.6, 2.1, 1.9, 2.6, 3.4, 3.5, 2.8, 3.4, 2.6, 1.9 TTR (by numerical average) <50%, F = 30 days (data not shown), M = 2.45, N = 20, R = 1.1–3.5, X_1_ = 30% and X_2_ = 12.5%

Abbreviations: F: average time between INR checks; M: mean of all INRs; N: number of INR measurements; R: range of INR values, X_1_: percentage of INRs <2.0; X_2_: percentage of INRs >3.0
